# The Eczema Bathing Study: Weekly versus daily bathing for people with eczema? Protocol of an online, randomised controlled trial

**DOI:** 10.3310/nihropenres.13659.1

**Published:** 2024-10-14

**Authors:** Wei Chern Gavin Fong, Laura Howells, Ingrid Muller, Eleanor J Mitchell, Arabella Baker, Leila Thuma, Eleanor Harrison, Lucy Bradshaw, Yimin Jiang, Fiona Cowdel, Paul Leighton, Alan Montgomery, Jane Ravenscroft, Matthew J Ridd, Miriam Santer, Reiko J Tanaka, Nicholas Hilken, Richard Swinden, Richard Dooley, Carron Layfield, Clare Upton, Sophia Collins, Firoza Davies, Tracy Owen, Mars Eddis-Finbow, Devin Patel, Goldie Putrym, Hywel C Williams, Amanda Roberts, Kim S Thomas

**Affiliations:** 1 Centre of Evidence Based Dermatology, Lifespan and Population Health, University of Nottingham School of Medicine, Nottingham, England, UK; 2 Primary Care Research Centre, University of Southampton, Southampton, England, UK; 3 University of Nottingham Nottingham Clinical Trials Unit, Nottingham, England, UK; 4 Birmingham City University Faculty of Health Education and Life Sciences, Birmingham, England, UK; 5 Centre for Applied Excellence in Skin & Allergy Research, University of Bristol Centre for Academic Primary Care, Bristol, England, UK; 6 Department of Bioengineering, Imperial College London Faculty of Engineering, London, England, UK; 7 Citizen Scientist contributor, England, UK

**Keywords:** Atopic dermatitis, Bath, Eczema, Evidence, Personal care, Randomised controlled trial, Shower, Skin care, citizen science

## Abstract

**Background:**

A priority setting partnership for eczema (syn atopic eczema, atopic dermatitis) has identified that bathing frequency is a key area of patient interest. However, there are nolarge, high-quality randomised controlled trials (RCTs) investigating this.

The Rapid Eczema Trials project is a novel programme of research that aims to deliver multiple online RCTs, using a citizen science approach. This project involves working with members of the public to co-design and conduct studies that answer questions of importance to them. The first trial to be conducted through this project is assessing the impact of bathing frequency on eczema.

**Methods:**

This is an online, two-arm, parallel-group superiority RCT with internal pilot phase. People aged ≥1 year with eczemaliving in the United Kingdom are eligible. Exclusion criteria are: people with other types of eczema such as venous eczema, hand eczema and contact eczema; recently started a new eczema treatment; taking part in another eczema trial; Patient Oriented Eczema Measure (POEM) ≤2; planning to swim more than twice a week; unable/unwilling to change bathing practices. Participants are allocated 1:1 to either the weekly bathing group (bathe 1 or 2 times a week) or the daily bathing group (bathe 6 or more times a week) for 4 weeks. The primary outcome is POEM, assessed weekly over 4 weeks. Secondary outcomes include skin specific quality of life, eczema control, itch severity, use of usual eczema treatments, proportion who achieve an improvement in POEM of ≥3 points, global change in eczema and safety outcomes. A sample of participants will also be invited to a semi-structured interview to discuss their experience. The primary comparative analysis will be according to randomised allocation regardless of actual frequency of bathing. The trial will be reported in accordance with CONSORT guidelines. The study has received ethical approval by the London - Surrey Research Ethics Committee (2 Redman Place, London, E20 1JQ, United Kingdom) on 11/10/2023 ( approval number: 23/PR/0899).

**Trial registration:**

ISRCTN12016473, 22/11/2023, https://doi.org/10.1186/ISRCTN12016473

## Plain Language Summary

This study is part of the Rapid Eczema online Trials project which aims to answer questions about the self-management of eczema. People with eczema are helping to plan and run all parts of these studies.

In the Eczema Bathing Study, we’re finding out how often people with eczema should have a bath or a shower to best manage their eczema. At the moment, we don’t know the answer to this question. Some people believe that daily bath sare important, while others think it might dry out their skin too much. Anyone aged 1 year or older with eczema can join the study by signing up on the study website. We are encouraging people from all different ethnic and socioeconomic backgrounds to take part, to ensure the study is representative and can be applied to the general population of the United Kingdom (UK).

Participants will be asked about their eczema and how they usually look after their skin. They will be asked to have a bath or shower once or twice a week (Weekly bathing group), or to have a bath or shower 6 or more times a week (Daily bathing group). It will not be possible for people to choose which group they are in as this is decided by a computer. This is important to make sure that the two groups of people are similar. Any differences between the groups are then likely to be due to the different ways of bathing.

People in the study will be asked to follow the advice about how often to have a bath or a shower for 4 weeks. They will complete some questions every week. People can take part from home.

When the study results are known, we will share the results as quickly as possible on the study’s website and social media ( www.RapidEczemaTrials.org).

## Background and rationale

Eczema (syn atopic eczema, atopic dermatitis) is an itchy, chronic skin condition with significant impact on quality of life ^1^. Questions about skincare, washing and how to use eczema treatments are a high priority for patients, as reflected in the 2013 James Lind Alliance Priority Setting Partnership (PSP) ^2^. However, these topics are rarely the focus of large, high-quality randomised controlled trials (RCTs) ^3^. Similarly, a crowd sourcing prioritisation exercise by the Global Parents of Eczema Research initiative, using natural language processing of online social media posts ^4^, confirmed ongoing debate in these topics.

A systematic review of international eczema guidelines ^5^ highlights variability in the recommendations surrounding bathing practices, driven by lack of robust evidence to inform practice. A systematic review in 2020 included 16 prospective studies on bathing and eczema ^6^. Although this systematic review highlighted that increased frequency of bathing was not associated with worsening of eczema, most of the studies were not RCTs, or did not compare different frequencies of bathing. It was also unclear how timing of emollients or topical corticosteroid application following bathing was accounted for in the results.

Only two studies in the systematic review were RCTs specifically designed to answer questions around bathing frequency. One RCT ^7^ compared twice weekly vs daily bathing in children with eczema. The study did not find differences in eczema severity between the two. Conversely, another study which compared twice daily bathing versus twice weekly bathing found that twice daily bathing resulted in a greater reduction of eczema severity ^8^. However, these two trials both had small sample sizes (<50 participants), a short follow up (2 weeks) and the latter trial did not include people with mild eczema. Thus, the evidence surrounding bathing, including the optimal frequency of bathing is still unclear.

Efficient methodologies are required to address patient priorities around the self-management of eczema in a timely and resource efficient manner, whilst providing robust evidence to inform self-care choices. The Rapid Eczema Trials project was created for such a purpose ^9, 10^. Overall, this project aims to improve the lives of people living with eczema by working with citizen scientists to deliver multiple, efficient, online RCTs and share new knowledge with those who need it.

This research project aligns with the UK Government’s recent strategy document on the Future of UK Clinical Research Delivery ^11^, which calls for patient-centred research that is “streamlined, efficient and innovative”, and supports the UK Government’s agenda for reducing health inequalities in healthcare and research.

The Rapid Eczema Trials project aims to deliver multiple RCTs that answer questions about the self-management of eczema. This protocol describes the first of these trials, called the Eczema Bathing Study.

## Protocol

### Patient and Public Involvement

This trial is part of the Rapid Eczema Trials project and has therefore been co-designed in partnership with members of the public (citizen scientists). The trial design co-production group determined the eligibility criteria, defined the intervention and comparator, selected the outcome measures, determined the study duration, developed the recruitment strategy and planned the process evaluation. Co-producing this trial took nine months, consisting of 13 online meetings (five to prioritise the topic, four to develop the intervention and four to design the trial), requiring 39 hours of time commitment from public members (n = 12). The project is co-led by a researcher (KT) and a patient with lived experience of eczema (AR). Citizen scientist partners highlighted the need to ensure that trial participants were not financially disadvantaged through taking part in the trial (e.g. if asked to have a bath or shower more often than normal), and that payment methods were handled with sensitivity and minimal paperwork. They also helped address challenges related to online consent processes for people under 16; helped to understand the advantages and disadvantages of translating materials into other languages versus using simple language accessible to non-native English speakers; and highlighted cultural differences in bathing practices. Citizen scientists helped prepare study materials and tested the trial database, ensuring that processes were streamlined, and materials were inclusive and accessible.

### Objectives

The aim of this trial is to explore the impact of bathing frequency on eczema symptoms, quality of life and disease control in children and adults with eczema. Specifically, the trial will:

1. Assess the impact of weekly bathing (1 or 2 times per week) compared to daily bathing (6 or more times per week) in people with eczema over 4 weeks.

2. Explore barriers and facilitators to changing bathing practices and to understand the impact of trial processes on trial participation.

### Study design

This trial is a multicentre, online, two-arm, parallel group, superiority RCT, with an internal pilot phase to test key feasibility parameters. Each participant will be in the trial for 4 weeks with weekly outcome assessments. The pilot phase will end once 20% of the target sample size has been recruited or after 4 months, whichever happens first.

### Study setting

This is an online trial with no face-to-face assessments. Participants can join the study from anywhere in the UK.

### Eligibility criteria

***Inclusion criteria***

• Aged ≥1 year with self-report of eczema

• Usual residence in the UK

• Able and willing to give informed consent (or parent/legal guardian able and willing to give informed consent for children under 16 years)

***Exclusion criteria***

• None or very mild eczema symptoms (Patient Orientated Eczema Measure score ^12^ [POEM] ≤2)

• Eczema only present on hands (likely to be hand eczema or contact dermatitis); limited to locations where skin exposed to nickel e.g. jewelry (likely to be contact dermatitis); or eczema only around varicose veins (likely to be varicose eczema)

• Started a new eczema treatment (including antibiotics for eczema) other than emollients in the last 4 weeks

• Taking part in another eczema intervention trial

• Unable or unwilling to change bathing practices for 4 weeks

• Planning to swim more than twice a week in the next 4 weeks (including surfing, scuba diving etc.)

• Member of household already participating in this trial

### Interventions

***Intervention group:*** weekly bath or shower (1 or 2 times a week).

***Control group:*** daily bath or shower (6 times or more a week).

An online survey to explore usual bathing practices in people with eczema (n = 169) informed the choice of intervention and control groups. As most people with eczema reported having a bath or shower daily, this was chosen to be the control group. The intervention and control groups were defined in the trial design meetings, and chosen to ensure sufficient separation between the groups.

Following randomisation, participants are provided with instructions detailing how often they should bathe according to their allocation for the next four weeks. Discussion in the co-production groups with citizen scientists determined that 4 weeks was about the maximum that people with eczema would be willing to persevere with a change in their bathing habits. It was also agreed that this duration limits financial burden and was determined to be of sufficient duration to detect an effect. Instructions have been designed to be accessible (including pictures and simple language) and inclusive (available in different formats). Examples of this can be found in the extended data of the online repository.

Participants are asked not to change any of their other bathing practices e.g. method of bathing, product use, water temperature etc. Participants are advised they can wash their face and body using a flannel/sponge in the sink in between showers or baths, and can wash their hair in between showers or baths.

For both groups, participants may use their usual eczema treatments (e.g. emollients and flare control creams) whenever they need to as per usual practice. They are asked not to change their usual eczema treatments (or to start a new treatment) during the trial, if medically possible.

Financial assistance of a single payment of £20 either by bank transfer or voucher. is offered to anyone who feels that the changes in recommended bathing practices might have increased their household costs.

### Outcomes

This trial includes the core outcome set (COS) measurement instruments for the patient-reported outcome domains, as developed by Harmonising Outcome Measures for Eczema ^13^ (HOME). Since all trials will be completed online, it will not be possible to assess the clinical signs core domain in the HOME COS. The outcomes were chosen by the co-design group and the frequency of data collection was also informed by co-design group to minimise participant burden.

***Primary outcome***

Eczema symptoms assessed weekly over 4 weeks by the Patient Oriented Eczema Measure (POEM) ^12^. POEM consists of 7 items, scored 0 to 28 with higher scores representing more severe eczema.

***Secondary outcomes***

- Itch intensity (Peak Pruritis Numerical Rating Scale ^14^ (NRS) 24- hour peak itch) - one item, scored 0 to 10. Assessed at baseline and 4 weeks.
- Eczema control (Recap of atopic eczema, RECAP ^15^) – 7 items, scored 0 to 28. Assessed at baseline and 4 weeks.
- Skin-specific quality of life (Infants' Dermatitis Quality of Life Index (IDQoL ^16^) (under 4 years), Children’s Dermatology Life Quality Index (CDLQI ^17^) (from 4 years to 15 years) or Dermatology Life Quality Index (DLQI ^18^) (16 years and over) depending on age) – 10 items, scored 0 to 30. Assessed at baseline and 4 weeks.
- Use of usual eczema treatments assessed weekly over 4 weeks:
  - Number of days in the last week flare control creams (topical corticosteroids or calcineurin inhibitors) used – this outcome will be used as an indication of days with eczema flares ^19^.
  - Number of days in the last week moisturisers (emollients) used.
- Proportion of participants who achieve an improvement in POEM at week 4 of ≥3 points compared to baseline ^20^.
- Global change in eczema compared to baseline. Assessed at week 4.
- Safety outcomes
  - Change in eczema treatments
  - Sought advice from a health care provider as a result of a worsening of the eczema.

For children under 16 years, proxy reporting by a parent or carer will be accepted.

Please see Table 1 for trial assessments planned.

Adverse events are not being collected for this trial. Changes in eczema treatments and healthcare contact due to worsening of eczema are collected at week 4.

### Progression criteria

The following criteria will be considered at the end of the internal pilot phase. Aspects that meet these milestones will be flagged. Remedial actions will be discussed and implemented with input from the wider Rapid Eczema Trials programme team and independent Programme Steering Committee. As this is a simple advice trial / behavioural intervention, with minimal safety implications, no Data Monitoring Committee was required.

Criteria:

Recruitment: < 20% of total sample size at 4 months
• Adherence:
  - Daily bathing group: > 25% of participants reported to have bathed/showered < 6 times per week for two or more of the follow-up weeks
  - Weekly bathing group: > 25% of participants reported to have bathed/showered >2 times per week for two or more of the follow-up weeks.
• Data completeness: <85% of participants with POEM scores at week 1 and <70% of participants with POEM scores at 4 weeks (for those who have reached this timepoint)

### Sample size calculation

The sample size for the trial is based on POEM scores assessed weekly for 4 weeks and is designed to detect a difference of 2.2 points in POEM scores between the two groups. A small difference of 2.2 points has been chosen as it is not anticipated that there will be large effects from a change in bathing frequency, but even small differences could be important for people looking for self-management options to try at home. This difference represents a small change that is likely to be beyond measurement error ^20^. Assuming a standard deviation in weekly POEM scores of 6.5 and a correlation between repeated measurements of 0.8 (based on data from previous eczema RCTs), a sample size of 156 participants per group is required to detect this difference with 90% power and 5% two-sided significance level. Allowing for 20% loss to follow-up, gives a total sample size of 390 participants.

## Recruitment

Participants are recruited through various online and offline methods including mailouts from general practices, social media advertising, eczema support charities and existing networks.

Participants identified through General Practitioner (GP) practices have a recorded diagnosis of eczema on their medical records and have been issued a prescription for emollients or topical corticosteroids in the last 2 years.

The INCLUDE framework ^21^ was used during trial development to explore barriers and facilitators to including people from diverse backgrounds in terms of ethnicity, socio-economic status and geographical location.

Potential UK participants are guided to the trial website ( www.RapidEczemaTrials.org), where they are provided with online information about the trial. Information is provided in engaging formats appropriate for all ages (including videos, infographics and downloadable pdf).

Recruitment started on 29 ^th^ January 2024 and will continue for up to 12 months. Recruitment is through a variety of online and offline channels, including:

- Database search and mailout or text messages from GP practices serving as participant identification centres
- Eczema Citizen Science Community: monthly newsletters sent to people on the Rapid Eczema Trials mailing list with encouragement to promote the trial via their personal networks using snowballing recruitment.
- Existing mailing lists of people with eczema: e.g. previous trial participants who provided their consent to be re-contacted.
- Social media: via paid advertisements on Facebook and Instagram (including targeting specific characteristics) and non-paid social media promotion through existing groups and using a variety of platforms (e.g. X, Reddit).
- Outreach and engagement events
- Internal communication channels of partner organisations and eczema charities (e.g. social media accounts, website, existing consented mailing lists and newsletters).
- NIHR Be Part of Research website ( https://bepartofresearch.nihr.ac.uk/)
- Posters and flyers: displayed in e.g. schools, clinical settings, community centres, grocery stores (with permissions from relevant staff members).

Participants are encouraged to contact the trial team if they have any questions prior to registering for the trial online or if they have any questions throughout the trial.

## Trial procedures

The trial is managed by the Nottingham Clinical Trials Units (NCTU).

Following an initial self-reported eligibility screening completed by the potential participant online, written electronic consent (e-consent) is taken prior to completion of trial procedures and questionnaires. For children aged less than 16 years, e-consent is provided by the parent/carer. In addition to providing e-consent, parents/carers are asked to confirm that they have discussed participation in the trial with their child (if appropriate) and that their child is willing to take part. Children under 16 years have the option to give their assent on the e-consent form.

If eligible for the trial and consent is provided, participants are asked to complete the POEM to determine eligibility. Participants with a POEM score of > 2 are asked to complete the following:

Demographic data, UK Diagnostic criteria, use of eczema medications, prior belief on the frequency of bathing and eczema symptoms, and usual bathing practices (e.g. usual temperature of the water, use of shampoo, use of emollient wash products, and application of emollients/flare control creams after bathing).
HOME Core Outcome Measurement Instruments - POEM, NRS 24-hour itch, RECAP and quality of life instruments.
Additional information to inform analysis and interpretation of the trial:
  - Number of times had a bath or shower in the previous week - assessed weekly over 4 weeks to evaluate adherence to allocated frequency of bathing routine.
  - Process outcomes: ease of bathing as allocated, willingness to continue bathing strategy, eczema treatment usage, things that helped or made it difficult to bathe as allocated, experience of being in the trial. Assessed at 4 weeks.

All assessments are carried out online through secure, bespoke weblinks to questionnaires sent via email or text. Participants are sent email and text reminders to complete their questionnaires.

For children unable to complete patient-reported outcomes themselves, proxy reporting by a parent/carer is accepted, but, where possible, participants are encouraged to complete patient-reported outcomes in discussion with their child. Parents/carers are advised that this should be the same adult throughout the trial if possible.

An email/text with a unique weblink to the questionnaires is sent to the participant/parent/carer. For weeks 1, 2 and 3, participants receive a maximum of 2 reminders by email/text for each questionnaire if it has not been completed. At the final 4-week timepoint, participants receive text, email or phone call reminders to complete the final follow-up questionnaires (for up to 2 weeks after the questionnaire is due).

Participants who complete questionnaires at week 4 are offered the chance to enter a free prize draw to win £25, a child-friendly book about eczema, or both, according to their preference.

**Figure 1.**
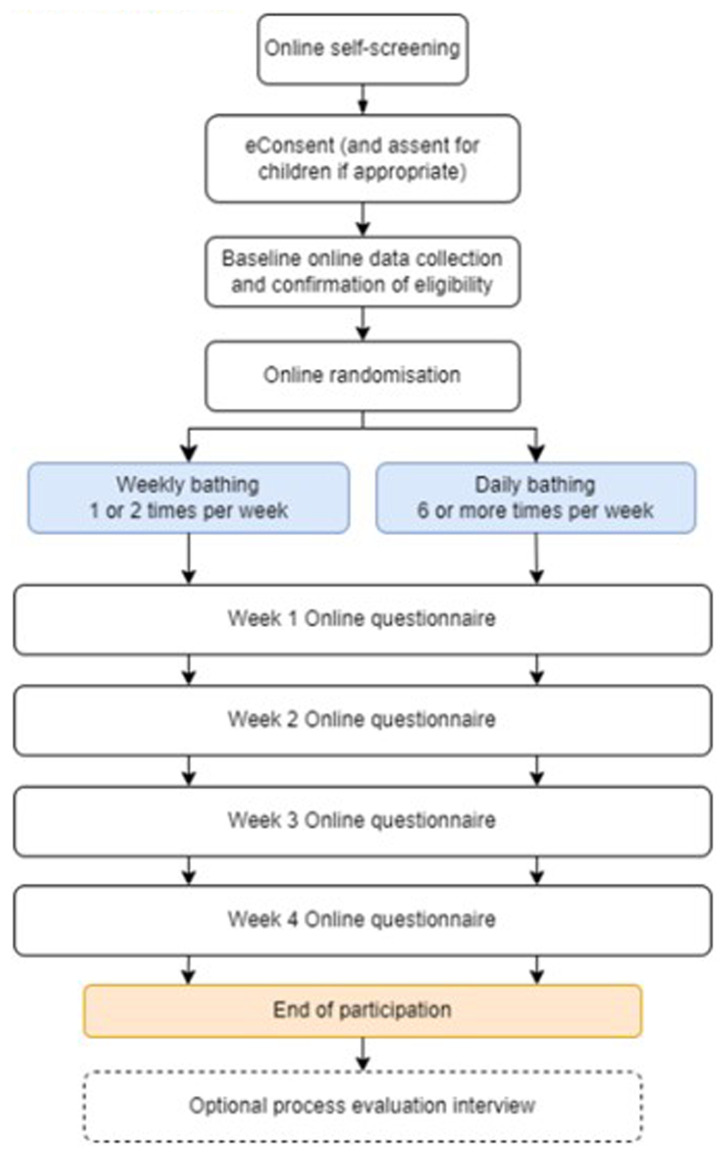
Trial flowchart.

A summary of assessments is shown in Table 1. The proposed trial flow chart is provided in Figure 1.

**Table 1.** Summary of trial assessments.

	TRIAL PERIOD					
	Enrolment	Randomisation	Follow-up			
TIMEPOINT	0	0	Week 1	Week 2	Week 3	Week 4
**ENROLMENT:**						
**Eligibility screen (including ** **self-report of eczema ** **diagnosis)**	X					
**Informed consent**	X					
**Eczema Symptoms (POEM) ** **– exclude if POEM ≤2**		X	X	X	X	X
**Minimisation variables**		X				
**Randomisation**		X				
**INTERVENTIONS:**						
**Weekly bathing (1 or 2 times ** **per week)**			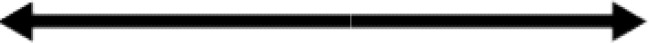			
**Daily bathing (6 or more times ** **per week)**			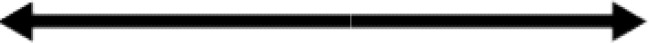			
**ASSESSMENTS:**						
Demographics and baseline characteristics		X				
UK Diagnostic criteria		X				
Usual bathing practices		X				
Prior belief in intervention		X				
Peak Pruritis Numerical Rating Scale (NRS)		X				X
Eczema Control (Recap of atopic eczema- [RECAP])		X				X
Quality of life (DLQI, CDLQI, IDQI as appropriate)		X				X
Use of eczema medications		X	X	X	X	X
Global change in eczema compared to baseline						X
Acceptability of intervention						X
Adherence to intervention			X	X	X	X
Adverse events – changes in eczema treatments and healthcare professional contact due to worsening of eczema)						X

POEM: Patient Orientated Eczema Measure; CDLQI: Children’s Dermatology Life Quality Index; DLQI: Dermatology Life Quality Index; IDLQI: Infants' Dermatitis Quality of Life Index.

The trial team monitor attempts to re-randomise the same individual or to enrol multiple people per household on an ongoing basis. Electronic forms collecting data for screening, consent and eligibility are recorded and processed with automatic and/or manual checks. These checks include ensuring identifiers and contact details are unique for participants, to avoid duplicate entries. Any potential duplicate participants that have registered with same email, phone number or postcode will be reviewed.

Any participants who do not provide consent during the recruitment process, prior to randomisation, are not randomised, and no further data is collected.

### Withdrawal procedures

Participants may withdraw their consent for follow-up and/or other trial related activities or receiving trial related communications.

As the trial is being conducted online with minimal contact with the research team, participants wishing to stop completing the questionnaires or stop their allocated intervention, will probably simply stop. In these cases, participants continue to receive links to the trial questionnaires until the end of their planned follow-up.

### Randomisation

Potential participants are randomised online once consent has been provided, they have submitted their baseline information, including the outcomes specified above, and eligibility confirmed.

Randomisation, data collection, data management is provided by a secure online system at NCTU: REDCap (Research Electronic Data Capture).

Participants are randomised 1:1 to either the intervention group (weekly bathing) or control group (daily bathing) using a minimisation algorithm with a probabilistic element balancing on the following factors:

• Eczema severity POEM score (3–7 mild, 8–16 moderate, 17–28 severe).

• Age (<4 years, 4–11 years, 12–15 years, 16–25 years, 26–55 years, >55 years)

• Usual method of bathing (bath or not bath)

The randomised allocated group will not be released to participants until after baseline data have been entered and stored on the trial database. Once this is done, their allocation is displayed on screen and the participant receives an automated e-mail or text to inform them of their allocation that includes links to the instructions.

### Blinding

Trial participants are aware of their randomised allocation as they may need to amend their bathing practices. Similarly, it is not possible to blind outcome assessments as this is an online trial with participant reported outcomes.

To mitigate the potential bias caused by lack of blinding, we will collect prior belief in the impact of bathing on eczema symptoms at baseline and explore this in a sensitivity analysis.

The trial statisticians, trial team at NCTU and members of the Trial Management Group will be blinded to treatment allocation until the database is locked, prior to analysis.

### Analysis

The analysis and reporting of the trial will be in accordance with CONSORT guidelines, with the primary comparative analyses conducted according to randomised allocation regardless of actual frequency of bathing. The primary analysis of the primary outcome will include participants with at least one weekly follow-up POEM score. The primary estimand for the trial is shown in Table 2. A statistical analysis plan will be finalised prior to database lock and release of the treatment allocations.

**Table 2.** Primary estimand.

Estimand component	Definition
**Population**	People with eczema aged 1 year and older
**Endpoint**	Eczema symptoms using the POEM assessed weekly assessed over 4 weeks
**Treatment conditions**	• Weekly bathing: no more than 1 or 2 times per week over a 4-week period • Daily bathing: 6 or more times per week over a 4-week period In both groups, participants will continue with usual medications and care to manage eczema (e.g. emollients, topical steroids etc)
**Population level summary estimate**	Difference in mean POEM score over 4 weeks between the two treatment conditions (weekly vs daily bathing)
**Intercurrent events**	
*Non-adherence to allocated group i.e. * *bathing frequency not as allocated*	Treatment policy – *all participant data included in analysis regardless of * *adherence with the allocated frequency of bathing.*
*Change to usual eczema treatments * *(including starting a new treatment) * *during the trial*	Treatment policy – *all participant data included in analysis regardless of whether * *there is a change in usual eczema treatment.*

The primary analysis will use all available longitudinal outcome data and will use a linear mixed effects model to estimate the difference in mean POEM score over the 4-week trial period with 95% confidence interval. The model will include fixed effects for the minimisation variables (age, baseline POEM score and usual method of bathing) as well as frequency of bathing, whether participants usually wash their hair in the bath/shower, whether they use emollient wash products, use of moisturisers and flare control creams after bathing, diagnosis of eczema according to the UK Diagnostic Criteria and whether participants are currently using systemic treatments. It will allow for observations nested within participants over time using random effects. If there is evidence of a differential effect over time, the difference in mean POEM score each week will be reported. Sensitivity analyses for the primary outcome will use multiple imputation for missing outcome data. Further supplementary analysis will investigate potential effects of compliance with allocated frequency of bathing to estimate the complier average causal effect (CACE). Participants will be considered as adherent if the number of times they report bathing/showering in the previous week is as per the allocated frequency of bathing strategy each week over the 4-week trial period.

Subgroup analyses for the primary outcome will be performed according to age at randomisation, usual method of bathing (bath/shower/other), diagnosis of eczema according to UK Diagnostic Criteria and prior belief on the frequency of bathing and eczema symptoms by including an appropriate interaction term in the mixed effect model.

The trial is not powered to detect any interactions hence the subgroup analyses will be treated as exploratory.

Between-group comparison of secondary outcomes will use an appropriate regression model for the outcome (linear for continuous outcomes, logistic for binary) with adjustment as described above for the primary outcome and baseline outcome measure for continuous variables if available. For secondary outcomes assessed weekly, mixed effects models will be used to allow for observations nested within participants over time using random effects.

### Process evaluation

A nested, qualitative interview study will investigate acceptability, feasibility, and adherence with regards to changed bathing practices. Interviews will also consider trial procedures. A purposive sample of existing trial participants who are willing to take part in a semi-structured interview will include participants of different ages, genders, ethnicities and prior beliefs in the impact of bathing on eczema symptoms and acceptability of the interventions. Equal numbers are being recruited from each randomised group of the trial (n = 15 to 20 per group). Only adults (aged 16+) are included in the process evaluation. E-consent is taken prior to the interview, and consent re-affirmed verbally at the start of the interview. Interviews are undertaken online or by telephone, at a time convenient to the participant, after the participant has completed their final follow-up assessment at four weeks. Interviews are digitally recorded if participants consent to this.

Interviews are exploring the participants’ (i) experience of changing bathing patterns; (ii) any barriers and facilitators to changing bathing patterns; (iii) any difference that this made to their eczema; (iv) their willingness to continue with their allocated bathing pattern; (v) their assessment of the effectiveness of the intervention and vi) their experience of taking part in the trial. Digital recordings are transcribed and anonymised. Recordings will be destroyed once transcripts have been approved as an accurate record. An inductive, thematic approach will be taken in analysing the data. This will develop a more detailed and contextualised understanding of the bathing intervention. A trained researcher is leading the analysis, with the support of PL.

### Data collection

All data are collected using the Research Electronic Data Capture (REDCap) ^22, 23^ platform. Data collection tools are usable on a range of digital devices, including smartphones.

### Data management and data entry

All data for the Rapid Eczema Trials will be via participant-report through electronic questionnaires and stored directly into the trial database. Participants’ medical records will not be accessed.

If the trial team become aware of any withdrawals or protocol deviations, these will be entered into REDCap by a member of the trial team.

Programmed validation and data quality rules will be used to identify data anomalies.

Checks will include missing data (including missing forms), out of range values, illogical entries and invalid responses.

Further data handling details can be found in the Rapid Eczema Trials Data Management Plan, which can be found in the extended data of the online repository.

### Data access

The Trial Statistician, Trial Manager and Data Manager will have access to the exported datasets via a secure log-in to the database to download the datasets.

Datasets are available in REDCap in realtime for a user with the appropriate permissions to download at any time.

Once all data has been locked, the final exported dataset in csv (raw) format will be subject to a quality control check to check numbers of records and fields are correct.

### Data monitoring and audit

All data are provided by participants in response to online surveys and are assumed to be source data for this trial with no additional data cleaning requirements. Data management is managed in accordance with an agreed data monitoring plan.

Since the Rapid Eczema Trials programme includes only low-risk trials, it was agreed with the sponsor and funder that a Data Monitoring Committee was not required. The Trial Master File and evidence of audits will be made available upon request for regulatory inspections.

### Confidentiality

Personal data recorded on all documents will be regarded as strictly confidential and will be handled and stored in accordance with the Data Protection Act 2018 and the UK General Data Protection Regulation (GDPR). All participants will be assigned a unique trial number at the time of randomisation. First names will be stored to allow personalised messages to individual participants when sending questionnaires and reminders. Protected characteristics (e.g. age, gender, ethnicity) are stored to enable monitoring of equality, diversity and inclusion data. Text messages and emails will be used to send participants reminders about trial procedures and to provide links to the required questionnaires using a personalised link. Participants who do not complete follow-up questionnaires will receive text/email/phone reminders by the trial team as appropriate. NCTU will maintain the confidentiality of all participant’s data and will not disclose information by which participants may be identified to any third party (except where this is required for trial purposes e.g. to send interventions or text reminders to participants) or organisations for which the participant has given explicit consent for data transfer (e.g. Laboratory staff, competent authority, Sponsor). Participants wishing to receive update newsletters will be added to the trial mailing list. All participants will be sent a summary of the results that will not include personal identifiers. Reports of qualitative data findings may include direct quotes from participants, but these will not be identifiable to individuals.

## Ethics and consent

The study has received ethical approval by the London - Surrey Research Ethics Committee (2 Redman Place, London, E20 1JQ, United Kingdom) on 11/10/2023 (approval number: 23/PR/0899). The trial was prospectively registered before it opened to recruitment: ISRCTN12016473; 22/11/2023. This study will adhere to the Declaration of Helsinki.

This paper is based on protocol: ISRCTN12016473_PROTOCOL_V2.0_31Oct23.

## Sponsor

The sponsor of this study is the Nottingham University Hospitals NHS Trust ( Researchsponsor@nuh.nhs.uk).

## Dissemination

Results of these trials will be submitted for publication in peer-reviewed journals.Results will be released as quickly as possible on completion of the trials, using lay-friendly formats. Trial participants and members of the Eczema Citizen Science Community will be sent a copy of the results. Summaries will be posted on the Rapid Eczema Trials website and shared with partner organisations and eczema charities. Prior to release, results will be quality checked according to the NCTU statistics standard operating procedure, and interpretation of the trial results will be discussed with members of the co-design groups, the Trial Management Group and the Trial Steering Group.

Since this is a citizen-science project, copies of the trial materials including the trial protocol, analysis plan, database code and analysis code will be made freely available on the Rapid Eczema Trials website for others to use. Academic journal manuscripts will be prepared by the research team and members of the coproduction groups and authorship will be determined by mutual agreement.

## Conclusion

In conclusion this trial is the first of several online trials which will be delivered through the Rapid Eczema Trials program. This novel approach will allow multiple trials to be delivered rapidly and efficiently using a master protocol, standardised analysis plan, and a template database that incorporates core outcome measurement instruments for eczema trials. The main strength of this trial lies in the fact that it has been co-designed with citizen scientists from wide backgrounds who have lived experience of eczema. Citizen science partners were involved in driving all key decisions over the design and conduct of the trial.

The strength of this approach has already been demonstrated by the trial progress to date. Within the first two months, over 50% of the target sample size was recruited. All progression criteria for the internal pilot phase were successfully met, suggesting that this co-produced trial was able to avoid many of the difficulties in trial recruitment and retention that are commonly experienced in RCTs ^24^. Other strengths of this study lie in the large sample size, stratified randomisation, use of validated outcome measures which are a part of the HOME core outcome set ^13^, pre-registration and the use of clearly defined estimands for the primary outcome.

The main limitation of this trial is that it is not possible to blind participants to their allocated intervention. However, the analysis will be adjusted to account for this by taking into account participants’ prior beliefs about bathing frequency.

It is hoped that this trial will provide robust evidence in a timely and efficient way, to answer uncertainty around the effect of bathing frequency on eczema symptoms. This topic has not been adequately addressed by research for many years, despite being an important question for people with eczema. The Eczema Bathing Study is paving the way for more trials delivered through this innovative citizen science approach.

## Data Availability

The datasets containing individual participant data analysed during the trial will be available upon request from the NCTU ( ctu@nottingham.ac.uk) a minimum of 6 months after publication of this main results paper. Access to the data will be subject to review of a data sharing and use request by a committee including the Chief Investigator and Sponsor and will only be granted upon receipt of a data sharing and use agreement. Any data shared will be pseudonymised which may impact on the reproducibility of published analyses. The protocol and statistical analysis plan are freely available on the trial registration website. FigShare. Project Title: Online Repository for Extended data relating to Rapid Eczema Bathing Study Protocol. DOI: https://doi.org/10.6084/m9.figshare.26093725.v1 ^25^ The project contains the following underlying data: 1. Data management plan 2. Interventional materials 3. Consent forms 4. SPIRIT checklist Figshare: SPIRIT checklist for The Eczema Bathing Study: Weekly versus daily bathing for people with eczema? Protocol of an online, randomised controlled trial. https://doi.org/10.6084/m9.figshare.26093725.v1 ^25^. Data are available under the terms of the Creative Commons Attribution 4.0 International license (CC-BY 4.0).
