# Supplementary material for: The Eczema Bathing Study: Weekly versus daily bathing for people with eczema? Protocol of an online, randomised controlled trial
Source: NIHR Open Res. 2024 Oct 14;4:63. [Version 1] doi: 10.3310/nihropenres.13659.1 (PMC11803374; doi:10.3310/nihropenres.13659.1)
Supplement: Peer review history

# Reviewer report: Reviewer response for version 1

**DOI:** 10.3310/nihropenres.14830.r34125

**Published:** 2025-01-09

**Authors:** Dorian Kern

**Affiliations:** 1 Karolinska Institutet, Stockholm, Sweden

I am grateful for the opportunity to review this manuscript. This is a well-planned randomised study with useful implications. I really appreciate the citizen-science approach and believe it is important to validate the kind of general advice that is usually given out in a variety of clinical contexts, but is not always well-researched. I believe the methodology to be solid and appropriate. I just have one specific concern:

 Schram et al. (2012) established the minimal clinically important difference (MCID) for POEM at 3.4 points. While I understand the current protocol uses a ≥3 point improvement to define responders ("proportion who achieve an improvement in POEM of ≥3 points"), consider aligning with the MCID by using ≥4 points. This adjustment provides a round number while exceeding the MCID threshold. If the authors prefer ≥3 points, I suggest including a brief justification in the Methods section.

 Referece:

 Schram  ME﻿, et al., 2012 [Ref 1]

 Minor: Typo second sentence in the abstract, "nolarge", under "methods" in the abstract, "eczemaliving"

Is the study design appropriate for the research question?

Yes

Is the rationale for, and objectives of, the study clearly described?

Yes

Are sufficient details of the methods provided to allow replication by others?

Yes

Are the datasets clearly presented in a useable and accessible format?

Yes

Reviewer Expertise:

Clinical psychology, medical psychology, intervention research

I confirm that I have read this submission and believe that I have an appropriate level of expertise to confirm that it is of an acceptable scientific standard, however I have significant reservations, as outlined above.

## References

[1] : EASI, (objective) SCORAD and POEM for atopic eczema: responsiveness and minimal clinically important difference. *Allergy* .2012;67(1) : 10.1111/j.1398-9995.2011.02719.x 99-106 10.1111/j.1398-9995.2011.02719.x 21951293

# Response

**Published:** 2025-01-15

**Authors:** Wei Chern Gavin Fong

**Affiliations:** CEBD, University of Nottingham School of Life Sciences, Nottingham, England, UK

Thank you for your review and your comments. Please see our response (##) to your comments.

**Comment**

**I am grateful for the opportunity to review this manuscript. This is a well-planned randomised study with useful implications. I really appreciate the citizen-science approach and believe it is important to validate the kind of general advice that is usually given out in a variety of clinical contexts, but is not always well-researched. I believe the methodology to be solid and appropriate. I just have one specific concern:**

**Schram et al. (2012) established the minimal clinically important difference (MCID) for POEM at 3.4 points. While I understand the current protocol uses a ≥3 point improvement to define responders ("proportion who achieve an improvement in POEM of ≥3 points"), consider aligning with the MCID by using ≥4 points. This adjustment provides a round number while exceeding the MCID threshold. If the authors prefer ≥3 points, I suggest including a brief justification in the Methods section.**

**Response**

 Thank you for your kind comments and your suggestion. The minimum important change for outcomes is never really a fixed value and will vary depending on the methods used in the MIC study, as well as the methods, study intervention and population of the study that it is being used to interpret.

 The MCID reported by Schram et al was based on a population of people recruited through secondary care who had mostly moderate or severe eczema.  The minimum important change (MIC) for POEM has since been explored in a variety of settings, and so we selected an MIC that was likely to be more representative of our target population and reflective of the fact that it is unlikely for frequency of bathing to have a large treatment effect.

 For the purposes of this study, we therefore used the Howells (2018) paper to estimate a meaningful change in POEM of 3 or more. This paper suggests a range of interpretations for different change in POEM scores:

**Change in the POEM** **Suggested interpretation**

 ≤ 2:                                          Unlikely to be a change beyond measurement error

 2·1–2·9:                                   A small change detected that is likely to be beyond measurement error                                                      but unlikely to be clinically important

 3–3·9:                                      Probably a clinically important change

 ≥ 4:                                          Very likely to be a clinically important change

 Here is the reference for your kind perusal:

 How should minimally important change (MIC) scores for the Patient Oriented Eczema Measure (POEM) be interpreted? A validation using varied methods. Howells L, Ratib S, Chalmers JR, Bradshaw L, Thomas KS; CLOTHES trial team. Br J Dermatol. 2018.

# Reviewer report: Reviewer response for version 1

**DOI:** 10.3310/nihropenres.14830.r34241

**Published:** 2024-12-24

**Authors:** Dr Heidi Singleton

**Affiliations:** 1 Bournemouth University, Bournemouth, UK

Is the rationale for, and objectives of, the study clearly described?

 Yes very clearly.

 Is the study design appropriate for the research question?

 Yes, the study design is appropriate for the objectives (I could not see a separate RQ).

 Are sufficient details of the methods provided to allow replication by others?

 Yes, though it would be useful to include details of any methods followed for the citizen science/PPIE approach.

 Are the datasets clearly presented in a useable and accessible format?

 This is a study protocol but they do intent to follow CONSORT reporting guidelines.

Report

 The submission is a very well written protocol for an RCT (with built in pilot and suitable power calculations) to compare daily showering/bathing with showering/bathing fewer times per week. The protocol has been co-designed with people with lived experience after extensive consultation with them.

 The team are international experts including an experienced person with lived experience.

 Title is appropriate and clear. Really interesting area of research, which could make a big impact on people living with eczema.

 In the abstract “nolarge” needs a space. And a space is needed between “eczemaliving".

 In correct space for “bath sare ” in the PLS.

 Interesting that the study included bathing or showering, it might have been possible to compare these variables also. Please do consider this in any amendments as it would be reasonably simple to design the study this way. A limitation would be in homes that do not have a shower or do not have a bath, adding in this dimension could make recruitment more challenging.

 Good to cite the James Lind Alliance Priority Setting Partnership. I agree it will be useful for a RCT to be used to find the answer to the question about bathing and impact on eczema.

 A strength is that this research is “patient centred”.

 Considering using PPIE rather than PPI.

 Excellent that patients can be reimbursed if they need to use more bathing emollients or hot water than usual. It is noteworthy that this took a long time to co-create with the citizens and will be beneficial for other researchers to take this into account when considering if they should follow a similar process (this is enough reason for including a little more detail or the method followed during the co-creation- citizen science). It appears that best practice EDI processes were followed in relation to considerations of language translation and cultural differences with bathing.

 Objectives are appropriate. It is a shame that there is no follow up beyond 4 weeks. Is it worth/possible to capture data beyond that, in relation to lasting impact? It might also be worth capturing (via a diary?) if there habits changed if they noticed a difference in their eczema, though this could be discussed in the interviews.

 Objective two is very interesting, for example what if a person goes running several times per week they might need to shower or bathe more often?

 The study design (intervention and control groups) is informed by a survey- this is another strength.

 You mentioned washing hair regardless of which group the participant is in. This will be key as some people (mainly women) with long hair might struggle to wash their hair without showering depending on the set up of their home and their mobility. You might want to collect data on length of hair and possibly even hair type as again this could impact how frequently someone would need to wash their hair.

 I think they need to record some details about how they are washing with a flannel/sponge in between e.g. did they fill a sink with water for a sponge down etc. This process could use more of less water and could use more or less friction etc. Will they be keeping a diary about this?

 Excellent recruitment strategy.

 Could ethnicity also be used in the randomization algorithm, and or gender, based on my comment about necessity of hair washing?

 Again please consider subgroup analysis for ethnicity and gender.

 There are several very old references, some of which I am sure can be replaced with more up to date ones.

 Overall, this is a really comprehensive protocol and an exciting study.

Is the study design appropriate for the research question?

Yes

Is the rationale for, and objectives of, the study clearly described?

Yes

Are sufficient details of the methods provided to allow replication by others?

Yes

Are the datasets clearly presented in a useable and accessible format?

Yes

Reviewer Expertise:

Eczema in children and young people. RCTs with nested qualitative element.

I confirm that I have read this submission and believe that I have an appropriate level of expertise to confirm that it is of an acceptable scientific standard.

# Response

**Published:** 2025-01-15

**Authors:** Wei Chern Gavin Fong

**Affiliations:** CEBD, University of Nottingham School of Life Sciences, Nottingham, England, UK

Many thanks for your review of our protocol. Please see our responses (##) to your comments below:

**Comment**

**The submission is a very well written protocol for an RCT (with built in pilot and suitable power calculations) to compare daily showering/bathing with showering/bathing fewer times per week. The protocol has been co-designed with people with lived experience after extensive consultation with them.**

**The team are international experts including an experienced person with lived experience.**

**Response**

 ## Thank you for your kind comment

**Comment**

**Title is appropriate and clear. Really interesting area of research, which could make a big impact on people living with eczema.**

**In the abstract “nolarge” needs a space. And a space is needed between “eczemaliving".**

**In correct space for “bath sare ” in the PLS.**

**Response**

 ## Thank you for spotting these typos, these have now been corrected

**Comment**

**Interesting that the study included bathing or showering, it might have been possible to compare these variables also. Please do consider this in any amendments as it would be reasonably simple to design the study this way. A limitation would be in homes that do not have a shower or do not have a bath, adding in this dimension could make recruitment more challenging.**

****

**Response**

 ##  As the reviewer rightly concluded, trying to add an additional layer of randomisation to explore bath versus shower would have added complications to this simple online study and would potentially have excluded people if they did not have access to both a bath and a shower.  It is also likely that people have strong feelings about which they prefer and would be less willing to join the study.

 As such, this notion was discussed with the co-production team at the time of designing the study, but was decided against.

 We are however collecting this information at baseline and have included method of bathing as part of the randomisation algorithm.  We also plan to conduct a sub-group analysis to explore the impact of method of bathing (bath or shower).

**Comment**

**Good to cite the James Lind Alliance Priority Setting Partnership. I agree it will be useful for a RCT to be used to find the answer to the question about bathing and impact on eczema.**

**A strength is that this research is “patient centred”.**

**Considering using PPIE rather than PPI.**

**Response**

 ## Thank you we have changed this

**Comment**

**Excellent that patients can be reimbursed if they need to use more bathing emollients or hot water than usual. It is noteworthy that this took a long time to co-create with the citizens and will be beneficial for other researchers to take this into account when considering if they should follow a similar process (this is enough reason for including a little more detail or the method followed during the co-creation- citizen science). It appears that best practice EDI processes were followed in relation to considerations of language translation and cultural differences with bathing.**

**Response**

 ## Thank you for your comment. Here is a reference to a recent paper our group has published regarding the co-production process.

 Baker A, Bonsu N, Howells, L et al (2025) Co-producing a randomised controlled trial on the frequency of bathing in eczema: description of a citizen science approach. *Skin Health and Disease (in press)*

**Comment**

**Objectives are appropriate. It is a shame that there is no follow up beyond 4 weeks. Is it worth/possible to capture data beyond that, in relation to lasting impact? It might also be worth capturing (via a diary?) if there habits changed if they noticed a difference in their eczema, though this could be discussed in the interviews.**

**Response**

 ## Unfortunately, this is not possible within the time and resources of the programme, but we will indeed explore these issues in the interview study.  The duration of follow-up of 4 weeks was chosen by our citizens science partners who felt that people would be unlikely to be willing to change their normal bathing practices for longer than 4 weeks as part of this trial.

**Comment**

**Objective two is very interesting, for example what if a person goes running several times per week they might need to shower or bathe more often?**

**Response**

 ## yes, this is the challenge of trying to conduct a pragmatic RCT and is the reason for allowing people to have a bath/shower 1-2 times per week, rather than just once per week to allow more flexibility in this regard.

**Comment**

**The study design (intervention and control groups) is informed by a survey- this is another strength.**

**You mentioned washing hair regardless of which group the participant is in. This will be key as some people (mainly women) with long hair might struggle to wash their hair without showering depending on the set up of their home and their mobility. You might want to collect data on length of hair and possibly even hair type as again this could impact how frequently someone would need to wash their hair.**

**Response**

 ## Unfortunately it is too late to add additional questions into the baseline questionnaire at this late stage, but we did collect information at baseline on whether people usually wash their hair in the bath/shower and adjusted for this in the primary analysis.

**Comment**

**I think they need to record some details about how they are washing with a flannel/sponge in between e.g. did they fill a sink with water for a sponge down etc. This process could use more of less water and could use more or less friction etc. Will they be keeping a diary about this?**

**Response**

 ## As this was a pragmatic, online trial with no contact between participants and the research team, we aimed to keep data collection to a minimum.  As such, we focussed on collecting adherence data most relevant to the primary question ie frequency of bathing. The interview study should hopefully help to draw out some of this additional information however.

**Comment**

**Excellent recruitment strategy.**

**Could ethnicity also be used in the randomization algorithm, and or gender, based on my comment about necessity of hair washing?**

**Again please consider subgroup analysis for ethnicity and gender.**

**Response**

 ## Thank you for We have not planned to conduct sub-group analysis based on ethnicity due to limited numbers in each ethnic group.  We can however present summary data by ethnicity in the trial report to allow for subsequent meta-analysis in the future.

 ## Thank you for your suggestion regarding sub-group analysis by gender, we will now include this in as an additional post-hoc subgroup analysis. 

**Comment**

**There are several very old references, some of which I am sure can be replaced with more up to date ones.**

**Response**

 ## Thank you for your comment. Having reviewed the manuscript again most of the very old references are references to the original papers for several of the patient reported outcome measures such as the POEM (2004), DLQI (1994), iDLQI (2001), cLDQI (1995).

**Comment**

**Overall, this is a really comprehensive protocol and an exciting study.**

**Response**

 ## Thank you for your kind and encouraging comment

# Reviewer report: Reviewer response for version 1

**DOI:** 10.3310/nihropenres.14830.r33742

**Published:** 2024-12-11

**Authors:** Ohya Yukihiro

**Affiliations:** 1 National Center for Child Health and Development, Okura, Tokyo, Japan; 2 Department of Occupational and Environmental health, Nagoya City University, Graduate School of Medical Sciences, Nagoya, Aichi, Japan; 3 Department of General Allergy, Fujit Medical School, Nagoya, Aichi, Japan

This trial addresses one of the important clinical questions such as the influence of bathing frequency for people with eczema on skin symptoms. The trial protocol is well-designed from an epidemiological point of view. However, I have a serious concern about the intervention methods that might influence the outcomes of this trial. 

 In the Interventions section, the authors described “Participants are asked not to change any of their other bathing practices e.g. method of bathing, product use, water temperature etc. “

 In particular, methods of bathing are critically important as well as bathing frequency for patients with eczema and the proportion of participants with some specific method of bathing might reverse the results of the trial. 

 For example, a large-scale birth cohort study in Japan revealed that a higher frequency of soap use when bathing resulted in a lesser prevalence of atopic dermatitis. (Ref 1)

 Prevalence of atopic dermatitis at 3 years of age was 6.9% in children with every time use of soap when bathing at 1.5 years of age, 9.8% in those with most time use, 14.5% in those with sometimes use, and 18.1% in those with seldom use. 

 These findings may be contrary to the common sense of Westerners. However, if you know the Japanese way of bathing, you can understand why. 

 Typical methods of bathing in Japanese are as follows: 

 1. Wash your whole body with soap outside the bathtub in the bathroom. 

 2. Completely wash out (not wipe out) the soap foam outside the bathtub by using plenty of warm water with showering or a washbowl.

 3. Soak your body in clean and warm water in the bathtub after washing out the soap. 

 4. Wipe out the water from your skin with a towel just after getting out of the bathtub.

 5. Apply emollient or moisturizer on the skin as needed. 

 This trial seems to be open to all ethnicities including non-native English speakers, although participants in this trial are limited to the usual residence in the UK. 

 Therefore, the influence of cultural differences in bathing methods on eczema should not be ignored. 

 To elucidate the influence of bathing frequency on eczema, a specific method of bathing should be fixed or a stratified analysis based on bathing methods classification is needed.

Is the study design appropriate for the research question?

Partly

Is the rationale for, and objectives of, the study clearly described?

Yes

Are sufficient details of the methods provided to allow replication by others?

Partly

Are the datasets clearly presented in a useable and accessible format?

Not applicable

Reviewer Expertise:

My clinical expertise is allergic diseases, especially atopic dermatitis, food allergies and asthma. My research expertise is epidemiology, especially clinical trials and cohort studies, concerning allergic diseases and behavioral sciences.

I confirm that I have read this submission and believe that I have an appropriate level of expertise to confirm that it is of an acceptable scientific standard, however I have significant reservations, as outlined above.

## References

[1] : Association of soap use when bathing 18-month-old infants with the prevalence of allergic diseases at age 3 years: The Japan Environment and Children's Study. *Pediatr Allergy Immunol* .2023;34(4) : 10.1111/pai.13949 e13949 10.1111/pai.13949 37102383

# Response

**Published:** 2025-01-15

**Authors:** Wei Chern Gavin Fong

**Affiliations:** CEBD, University of Nottingham School of Life Sciences, Nottingham, England, UK

Thank you for your review of our protocol paper. Please see our responses (##) to your comments.

**Comment:**

**This trial addresses one of the important clinical questions such as the influence of bathing frequency for people with eczema on skin symptoms. The trial protocol is well-designed from an epidemiological point of view. However, I have a serious concern about the intervention methods that might influence the outcomes of this trial.**

**In the Interventions section, the authors described “Participants are asked not to change any of their other bathing practices e.g. method of bathing, product use, water temperature etc. “**

**In particular, methods of bathing are critically important as well as bathing frequency for patients with eczema and the proportion of participants with some specific method of bathing might reverse the results of the trial.**

**Response:**

 ## We agree and is precisely why we asked people to “do what they normally do”, so that the only thing they were changing was the frequency at which they had a bath or shower.  

 We are collecting information at baseline about their usual method of bathing (bath, shower, other) and will use this as part of the randomisation minimisation algorithm to ensure balance between the groups. We also plan to conduct a sub-group analysis to explore the impact of different methods of bathing.

 See analysis section:

 “Subgroup analyses for the primary outcome will be performed according to age at randomisation, usual method of bathing (bath/shower/other), diagnosis of eczema according to UK Diagnostic Criteria and prior belief on the frequency of bathing and eczema symptoms by including an appropriate interaction term in the mixed effect model.”

**Comment:**

**For example, a large-scale birth cohort study in Japan revealed that a higher frequency of soap use when bathing resulted in a lesser prevalence of atopic dermatitis. (Ref 1)**

**Prevalence of atopic dermatitis at 3 years of age was 6.9% in children with every time use of soap when bathing at 1.5 years of age, 9.8% in those with most time use, 14.5% in those with sometimes use, and 18.1% in those with seldom use.**

**Response:**

 ## Thank you for sharing this interesting study.  Whilst these results are interesting, it is difficult to establish causality from an observational study such as this.  We did collect details of bathing products used by participants as part of the trial and adjusted for this in the primary analysis.

 Full details of what baseline variables were adjusted for are found in the analysis section:

 “The model will include fixed effects for the minimisation variables (age, baseline POEM score and usual method of bathing) as well as frequency of bathing, whether participants usually wash their hair in the bath/shower, whether they use emollient wash products, use of moisturisers and flare control creams after bathing, diagnosis of eczema according to the UK Diagnostic Criteria and whether participants are currently using systemic treatments.” 

**Comment:**

**These findings may be contrary to the common sense of Westerners. However, if you know the Japanese way of bathing, you can understand why.**

**Typical methods of bathing in Japanese are as follows:**

**1. Wash your whole body with soap outside the bathtub in the bathroom.**

**2. Completely wash out (not wipe out) the soap foam outside the bathtub by using plenty of warm water with showering or a washbowl.**

**3. Soak your body in clean and warm water in the bathtub after washing out the soap.**

**4. Wipe out the water from your skin with a towel just after getting out of the bathtub.**

**5. Apply emollient or moisturizer on the skin as needed.**

**This trial seems to be open to all ethnicities including non-native English speakers, although participants in this trial are limited to the usual residence in the UK.**

**Therefore, the influence of cultural differences in bathing methods on eczema should not be ignored.**

**To elucidate the influence of bathing frequency on eczema, a specific method of bathing should be fixed or a stratified analysis based on bathing methods classification is needed.**

**Response:** 

 ## Please see response above – a stratified sub-group analysis is planned.
